# Effect of *Helicobacter pylori* infection on the risk of acute coronary syndrome: A systematic review and meta-analysis

**DOI:** 10.1097/MD.0000000000018348

**Published:** 2019-12-16

**Authors:** Yizhen Fang, Chunming Fan, Huabin Xie

**Affiliations:** Xiamen University Affiliated Cardiovascular Hospital, Xiamen, Fujian, China.

**Keywords:** acute coronary syndrome, *Helicobacter pylori*, meta-analysis

## Abstract

**Background:**

Numerous studies have illustrated the association between *Helicobacter pylori* (*H pylori*) infection and acute coronary syndrome (ACS). However, the results are contradictory. Therefore, we conducted the meta-analysis to identify the association between *H pylori* and ACS.

**Methods:**

We performed a systematic search through electronic databases (Excerpta Medica Database, PubMed, Cochrane Library, and Web of Science). Pooled odds ratios (ORs) and 95% confidence intervals (CIs) were calculated with a random effect model. We also carried out the sensitivity analysis and publication bias.

**Results:**

Forty-four eligible studies involving 7522 cases and 8311 controls were included. The pooled result showed that *H pylori* infection was associated with an increase risk of ACS (OR = 2.03, 95% CI 1.66–2.47). In addition, similar results were obtained in subgroups of study quality, area, human development index, and *H pylori* detection method. The OR for developing countries was significantly higher than developed countries (OR = 2.58 vs OR = 1.69). Moreover, *H pylori* with cytotoxin-associated antigen A was also significantly associated with an increase risk of ACS (OR = 2.39, 95% CI 1.21–4.74).

**Conclusion:**

The meta-analysis suggested that *H pylori* infection was associated with an increased risk of ACS, especially in developing countries. *H pylori* is easily screened and can be treated with a wide range of drugs. Thus, more high-quality and well-designed studies are needed to confirm whether the treatment of *H pylori* is an effective way to reduce ACS risk.

## 1 Introduction

Acute coronary syndrome (ACS) is a common clinical syndrome of atherosclerotic progression in the coronary plaque, including unstable angina, non-ST-segment elevation myocardial infarction and ST-segment elevation myocardial infarction. Chronic inflammation is considered to be a possible causative agent in the development of ACS and local inflammation in the coronary artery wall may be involved in the pathogenesis of ACS.^[1]^ Nowadays, chronic bacterial infection, such as *Helicobacter pylori* (*H pylori*) infection and the accompanying inflammation were considered to be associated with onset of ACS.

*H pylori*, a spiral gram-negative bacterium, may cause a persistent low-grade inflammation. Several studies have shown that *H pylori* may contribute to the progression of atherosclerosis through chronic low-grade inflammatory stimulation.^[2,3]^ In addition, *H pylori* infection could increase risk of acute cardiovascular events by promoting atherosclerotic plaque instability or plaque disruption.^[4]^ Up to now, numerous studies have illustrated the link between *H pylori* infection and ACS. However, the sample sizes of these studies were limited, and the results are conflicting. Theses debatable conclusions leave the *H pylori* – ACS association studies under debate for many years. Thus, we carried out a meta-analysis to identify the association between *H pylori* and ACS.

## 2 Materials and Methods

The meta-analysis was performed according to the preferred reporting items for systematic reviews and meta-analyses checklist and followed these guidelines.^[5]^

### 2.1 Search strategy

A systematic search was performed through PubMed, Cochrane, Excerpta Medica Database (Embase) and Web of Science. The systematic search was updated on October 18, 2019. The following search terms were combined: “(“acute coronary syndrome” or ACS or “myocardial infarction” or “unstable angina” or “ischemic heart disease” or “coronary disease” or “myocardial ischemia” or “coronary atherosclerosis” or “sudden cardia death”)” and “(“*Helicobacter pylori”* or Helicobacter or “Helicobacter infection” or “*H. pylori”* or HP).” Language and publication year are not restrictive in our search.

### 2.2 Inclusion and exclusion criteria

Eligible studies should meet the following inclusion criteria:

(1) ACS as the outcome of study;
(2) evaluated the association between ACS and *H pylori*;
(3) presenting sufficient data to calculate odds ratios (ORs) and 95% confidence interval (CIs);
(4) case-control or cohort studies for human.

Exclusion criteria included:

(1) data deficiencies;
(2) duplicate publications;
(3) letter, comment, editorial, review, conference abstract;
(4) case-only study;
(5) no human study;
(6) no full text.

Two authors separately screened the potential studies according to above criteria. Disagreement was decided by the third author.

### 2.3 Data extraction

Two authors separately extracted the information from all eligible studies. Disagreement was resolved by the third author until all authors were unanimous. The following data were collected: name of first author, year of publication, country, area, number of cases and controls, human development index (HDI), matched variables, cytotoxin-associated antigen A (CagA), and so on.

### 2.4 Quality score assessment

All included studies were scored by 2 authors separately according to the predetermined criteria (Table 1) which were adjusted and revised from the Newcastle–Ottawa scale. Any divergence was decided by the third author. A study with quality score ≥7 was regarded as “high-quality study,” while a study with quality score <7 indicated “low-quality study.”

**Table 1:**
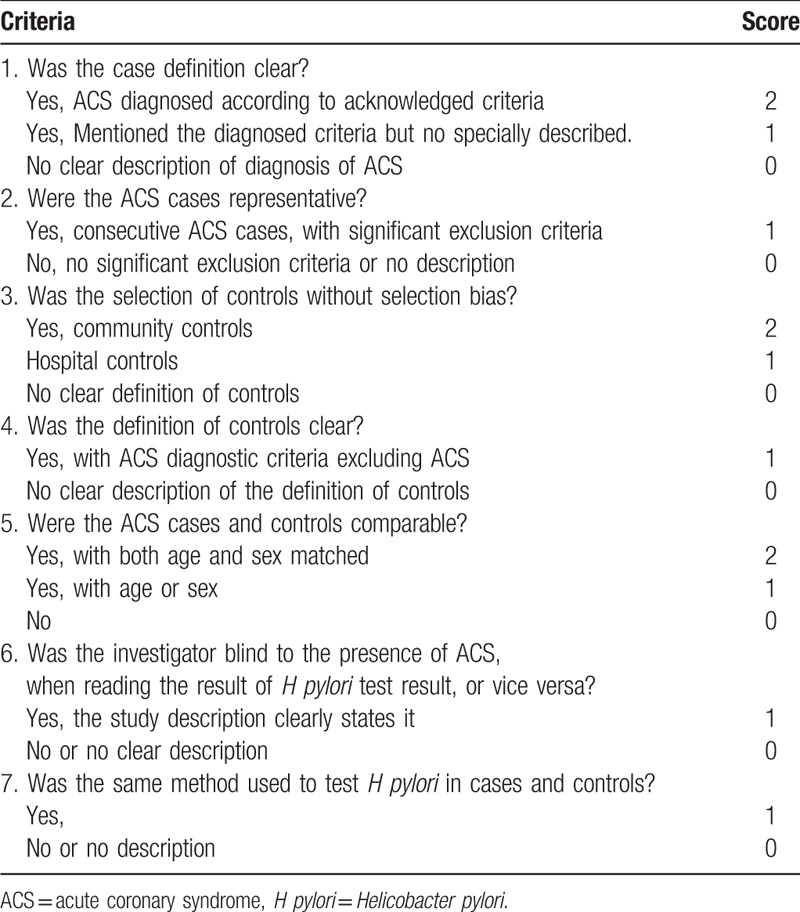
Quality evaluation tabulation.

### 2.5 Statistical methods

The prevalence data of *H pylori* infection (cases/controls) in each study were obtained. ORs and 95% CIs were calculated to assess the strength of the association between *H pylori* and ACS risk. Heterogeneity was assessed by the Q statistic (significant value at *P* < .1) and the *I*^2^ statistic (*I*^2^ > 50% indicating a significant inconsistency). When heterogeneity existed, we used a random-effect model (the DerSimonian and Laird method) to evaluate the pooled ORs and 95% CIs, otherwise, a fixed-effect model (Mantel–Haenszel method) was used. Sensitivity analysis was performed to evaluate the effect of omitting any single study. Egger test and Begg funnel plot were used to examine the publication bias (*P* < .05 suggested a significant bias). All analyses were performed in the STATA software (version 12.0; StataCorp, College Station, TX), with 2-sided *P*-values.

All analyses were based on previous published studies, thus no ethical approval and patient consent are required.

## 3 Results

### 3.1 Characteristics of studies

**Figure 1:**
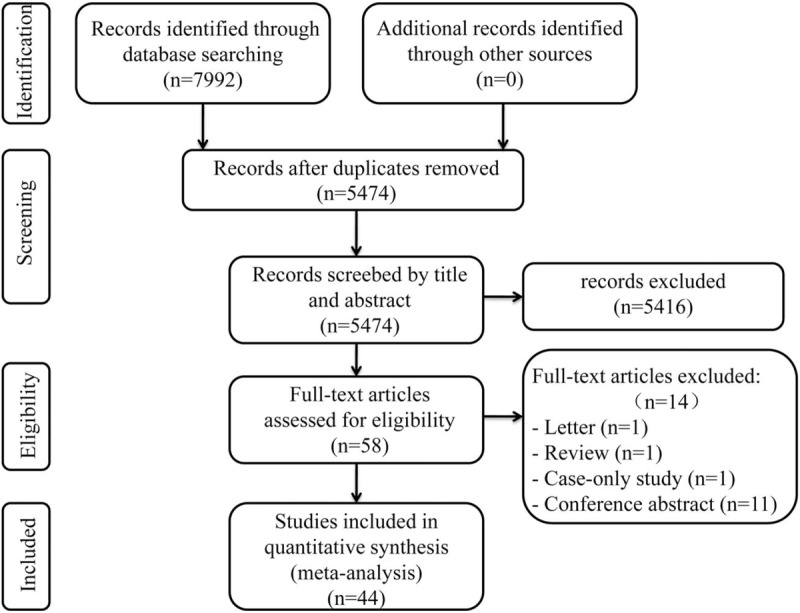
PRISMA flow diagram for study selection. PRISMA = preferred reporting items for systematic reviews and meta-analyses.

After the systematic search, a total of 7992 relevant studies were acquired from the PubMed, Cochrane, Embase, and Web of Science databases. Figure 1 shows the studies selection process. We removed 2518 duplicate studies and 5416 irrelevant articles. Then 14 studies were finally excluded, for the following reasons:

(1) 1 study was letter and 1 study was review;
(2) 1 study was case-only study;
(3) 11 studies were conference abstracts.

Finally, 44 eligible studies, publishing between 1996 and 2017, were included in this meta-analysis.

The meta-analysis included 7522 cases and 8311 controls. ACS patients had a higher rate of *H pylori* infection (64.49%, 4851/7522) than controls (48.03%, 3992/8311). The main features of included studies^[3,6–48]^ were shown in Table 2. Of these 44 studies, 27 studies indicated that *H pylori* infection was associated with an increased risk of ACS, while the others showed no association. Four of these studies were conducted in United Kingdom (UK), 1 in Croatia, 4 in Indian, 9 in Iran, 2 in Ireland, 9 in Italy, 4 in Japan, 1 in Macedonia, 1 in New Zealand, 2 in Pakistan, 1 in Spain, 1 in Sweden, 2 in Turkey, 2 in the United States, and 1 in China. UK contributed the most *H pylori* cases (9.95%) and the largest sample size (23.05%). The quality score for included 44 studies was ranged from 1 to 9, with 56.82% (25 of 44) of the studies being of high quality (score ≥7).

**Table 2:**
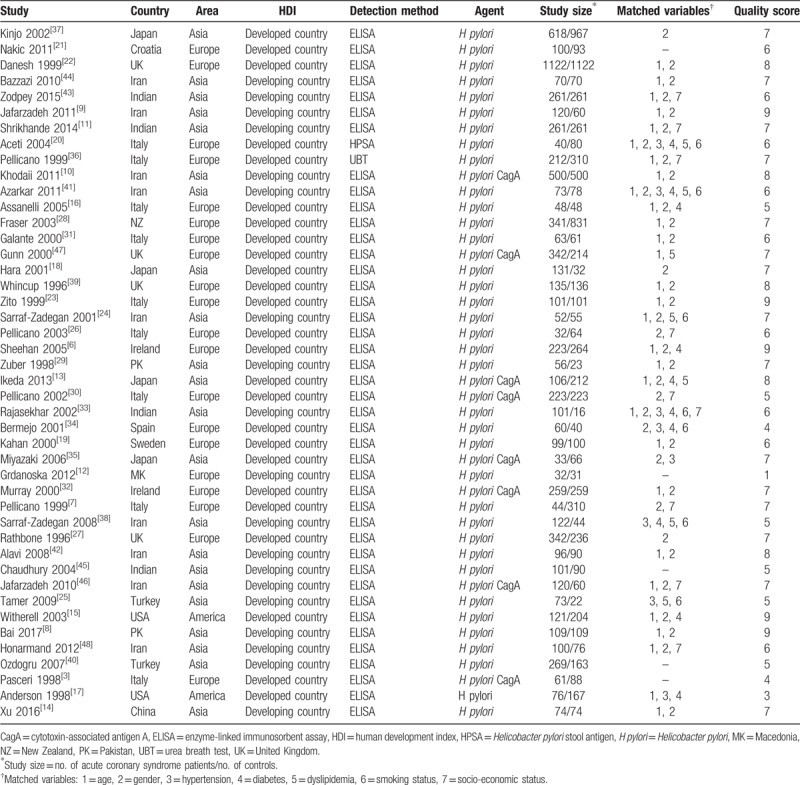
Characteristics of studies included in the meta-analysis.

### 3.2 Meta-analysis results

**Figure 2:**
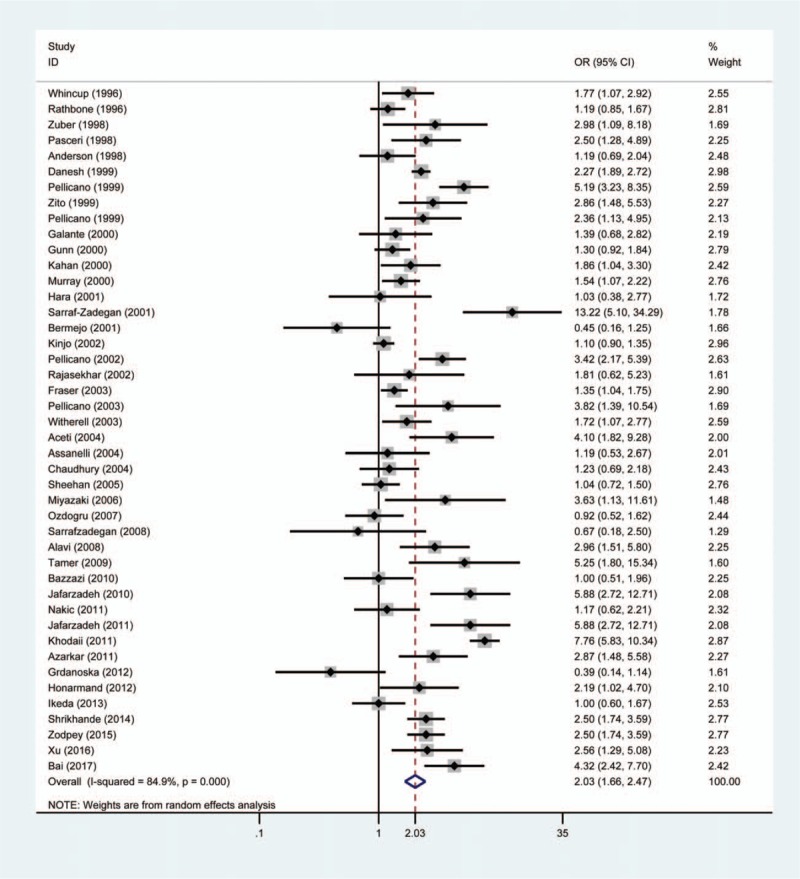
Forest plot of the association between *Helicobacter pylori* infection and acute coronary syndrome. The size of the black square represents the weight of the study in the meta-analysis. The rhombus represents the combined OR. OR = odds ratio.

The pooled result showed that patients with *H pylori* infection had a significantly increased risk of ACS compared with individuals without *H pylori* infection (OR = 2.03, 95% CI 1.66–2.47, *P*_*H*_ = .000) (Fig. 2). Moreover, studies with matched variables were chosen to perform another meta-analysis and the random effect pooled OR was 2.18 (95% CI 1.77–2.69, *P*_*H*_ = .000). Furthermore, we also conducted another meta-analysis which was based on different countries. A significantly positive association between *H pylori* and ACS was found in UK (OR = 1.60, 95% CI 1.11–2.29, *P*_*H*_ = .002), Iran (OR = 3.44, 95% CI 1.93–6.13, *P*_*H*_ = .000), Indian (OR = 2.20 95% CI 1.75–2.76, *P*_*H*_ = .168), Italy (OR = 2.80, 95% CI 2.03–3.86, *P*_*H*_ = .036), Pakistan (OR = 3.95, 95% CI 2.40–6.52, *P*_*H*_ = .532), and United States (OR = 1.47, 95% CI 1.03–2.10, *P*_*H*_ = .311). However, no statistically significant association was observed in Japan (OR = 1.13, 95% CI 0.94–1.35, *P*_*H*_ = .245), Ireland (OR = 1.27, 95% CI 0.98–1.64, *P*_*H*_ = .140), and Turkey (OR = 2.07, 95% CI 0.38–11.34, *P*_*H*_ = .005).

### 3.3 Subgroup analysis

**Figure 3:**
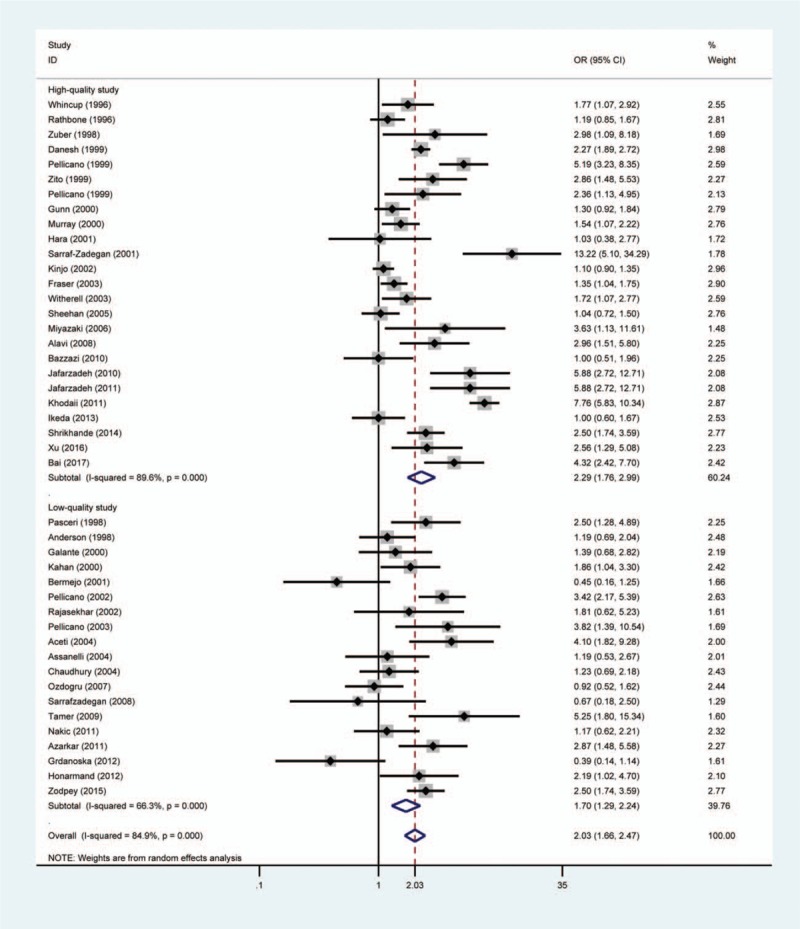
Forest plot of the association between *Helicobacter pylori* infection and acute coronary syndrome in the subgroup of study quality. The size of the black square represents the weight of the study in the meta-analysis. The rhombus represents the combined OR. OR = odds ratio.

**Figure 4:**
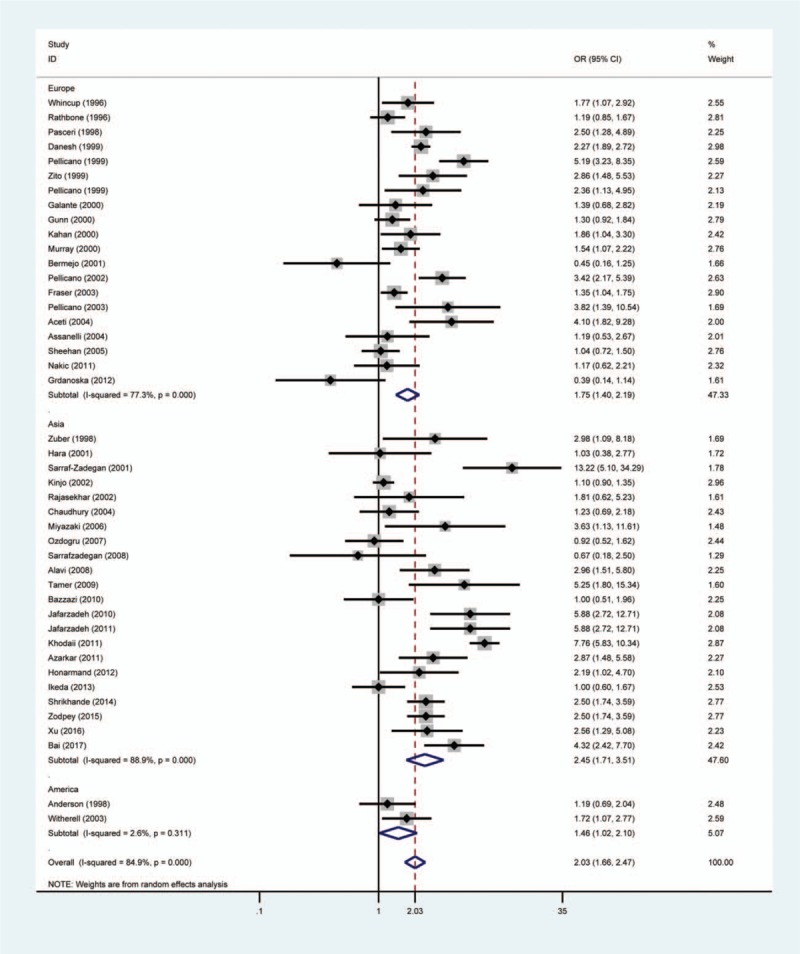
Forest plot of the association between *Helicobacter pylori* infection and acute coronary syndrome in the subgroup of geographical location. The size of the black square represents the weight of the study in the meta-analysis. The rhombus represents the combined OR. OR = odds ratio.

**Figure 5:**
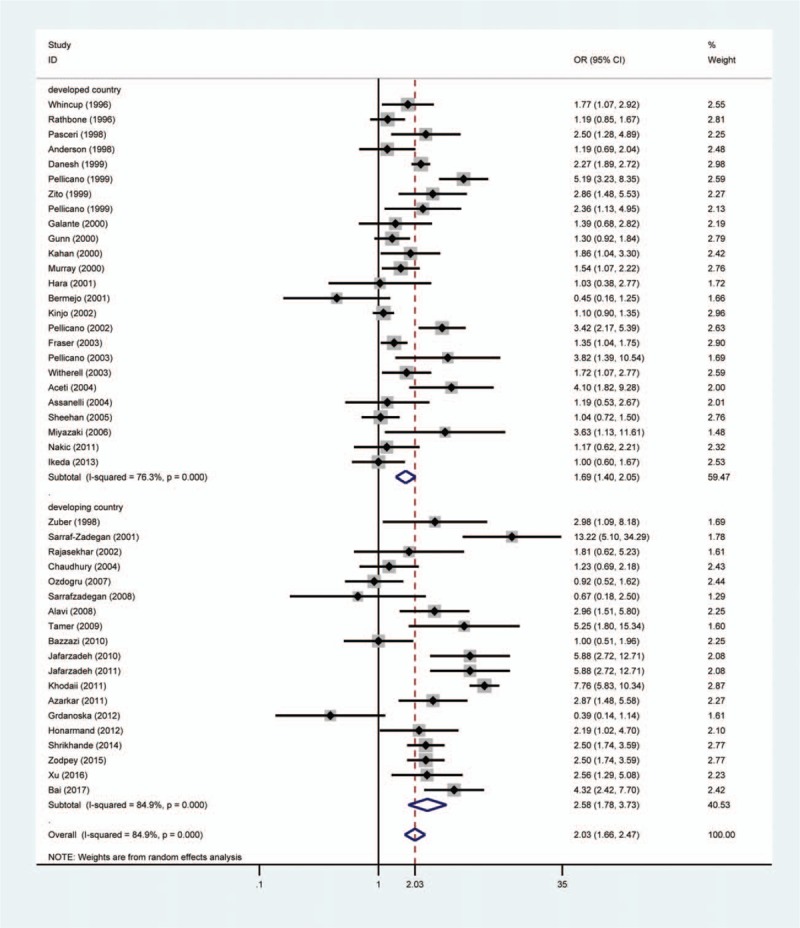
Forest plot of the association between *Helicobacter pylori* infection and acute coronary syndrome in the subgroup of human development index. The size of the black square represents the weight of the study in the meta-analysis. The rhombus represents the combined OR. OR = odds ratio.

Subgroup analysis was carried out to investigate the effects of study quality, area, HDI and *H pylori* detection method. There was significant association between *H pylori* infection and ACS risk in high-quality studies (OR = 2.29, 95% CI 1.76–2.99, *P*_*H*_ = .000) and low-quality studies (OR = 1.70, 95% CI 1.29–2.24, *P*_*H*_ = .000) (Fig. 3). In addition, we found a significantly positive association between *H pylori* and ACS in studies from Europe (OR = 1.75, 95% CI 1.40–2.19, *P*_*H*_ = .000), Asia (OR = 2.45, 95% CI 1.71–3.51, *P*_*H*_ = .000), and America (OR = 1.46, 95% CI 1.02–2.10, *P*_*H*_ = .311) (Fig. 4). A stronger association between ACS and *H pylori* was observed in developing countries than in developed countries (OR = 2.58, 95% CI 1.78–3.73 vs OR = 1.69, 95% CI 1.40–2.05) (Fig. 5). *H pylori* infection was assessed by the enzyme-linked immunosorbent assay (ELISA) in 95% included studies (42/44) and 2 other studies used ^13^C-urea breath test and *H pylori* stool antigen test to determine *H pylori* infection, respectively. A significant result was observed in the ELISA subgroup when stratifying findings by *H pylori* infection measure method (OR = 1.95, 95% CI 1.60–2.37, *P*_*H*_ = .000).

In 8 included studies, researchers focused on the association between CagA-positive strains of *H pylori* and ACS. A positive relationship was observed in 6 studies; however, 2 other studies revealed that there was no significant relationship between CagA strains infection and ACS. The random pooled result suggested an OR of 2.39 (95% CI 1.21–4.74, *P*_*H*_* *= .000).

### 3.4 Sensitivity analysis and publication bias

**Figure 6:**
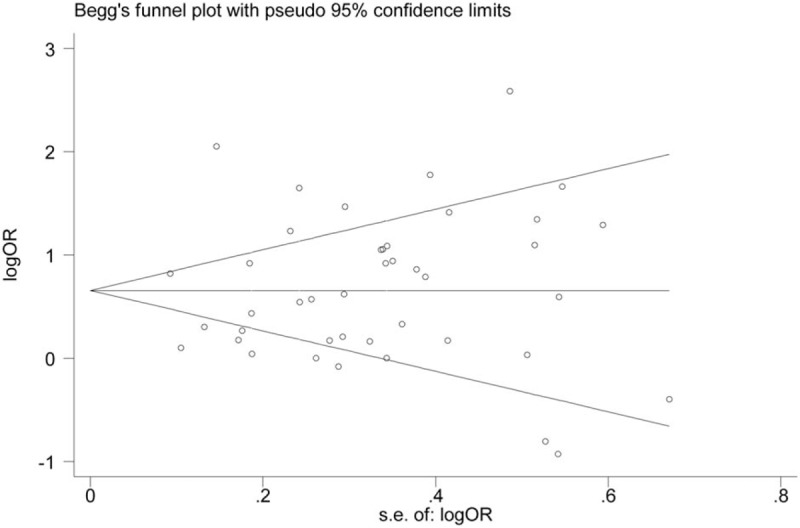
Begg funnel plot showing the publication bias analysis.

We detected the effect of individual studies on the pooled OR by sensitivity analysis. The pooled OR was not significantly altered after excluding 1 study at a time. Publication bias was assessed by the Begg funnel plot and Egger test. The results showed that no publication bias could be detected in the meta-analysis (*P*-value of Egger test:.560, *P*-value of Begg test:.317). Figure 6 shows the Begg funnel plot.

## 4 Discussion

*H pylori* has been proven to be the main pathopoiesis factor of various gastrointestinal diseases and the prevalence rate of *H pylori* infection was almost 48.5% around the world. Nowadays, many studies focus on the relationship between *H pylori* and ACS. However, the relationship of *H pylori* infection with ACS is still controversial. Our article including 15,833 participants is the first published meta-analysis to identify the association between *H pylori* and ACS. Based on the results from our meta-analysis, a significant higher prevalence of *H pylori* infection was found in ACS individuals than controls (64.49% vs 48.03%), which indicated a possible link between ACS and *H pylori*. The pooled result showed that individuals with *H pylori* infection have twice the risk of developing ACS. Sensitivity analysis demonstrated that any single study could not significantly influence the pooled result. Thus, all included studies had a good homogeneity and the pooled result was stable. No publication bias was found in this meta-analysis, which indicating that the pooled result was more reliable.

A positive association between *H pylori* and ACS was found in the high quality subgroup and low-quality subgroup, which was similar to the pooled result. In addition, 77.27% of included studies achieved satisfactory scores in the quality score assessment (≥6 scores) and more than half of studies were high-quality study. Therefore, the meta-analysis gave us reliable results, especially in the high quality subgroup. The meta-analysis demonstrated that the relationship between *H pylori* and ACS was stronger in developing countries than in developed countries (OR = 2.71 vs OR = 1.65). On one hand, the early poverty life was an important predicting factor of *H pylori* infection and the global prevalence rate of *H pylori* is 44.3%, ranging from 50.8% in developing countries to 34.7% in developed countries.^[49]^ One the other hand, *H pylori* may contribute to the progression of ACS in many ways, such as promoting inflammation reaction, increasing LDL-C levels and so on. Developed countries, with better living standards and medical care, could take effective intervention to reduce *H pylori*-associated adverse reactions, such as using lipid-lowering medicine, anti-inflammatory drug, and so on, which may weaken the role of *H pylori* in the progress of ACS. Moreover, the prevalence of *H pylori* was lower in Europe than in Asia from 1970 to 2006.^[50]^ The subgroup analysis of area showed that the link between ACS and *H pylori* was weaker in Europe than in Asia. All above further confirmed the positive correlation between *H pylori* and ACS. The result of breath test and *H pylori* DNA determination in stool might be affected by the proton pump inhibitors use and antibiotic use. However, the ELISA was used as a diagnosing test for *H pylori* infection in almost all included studies (95%, 42/44). Thus, we minimized these potential biases and the result of ELISA subgroup was consistent with the pooled result.

It is well known that many potential confounders are considered as risk factors for ACS and *H pylori* infection, such as age, gender, hypertension, smoking, socio-economic status, and so on. In the meta-analysis, 39 included studies considered the influences of these potential confounders. Thus, another meta-analysis including studies with matched variables was performed. The pooled result also showed a positive association between ACS and *H pylori*. It was suggested that these potential confounders were equally distributed between ACS patients and control individuals. Therefore, it was indicated that the pooled result was not affected by these confounding factors.

The meta-analysis also proved that the significantly increased risk of ACS could be caused by the CagA-positive strains of *H pylori*. The bacteria with a cytotoxin-associated protein could become more pathogenic and stimulated a heightened inflammatory response.^[51]^ CagA-positive *H pylori* could produce more cytokines which were transported in the blood-stream and enhance an inflammatory response in arteries. Furthermore, systemic markers of inflammation and acute phase reactants have been shown to be associated prospectively with ACS risk, and part of the beneficial effects of aspirin on cardiovascular disease risk appeared to be directly related to its anti-inflammatory.^[52]^ Thus, it is possible that CagA-positive *H pylori* infection may increase the risk of ACS through the induction of a more severe inflammatory response.

*H pylori*, the only microbial species surviving in the human stomach, is a gram-negative bacteria. *H pylori* infection is commonly acquired in early age and lasted a life-time. Nowadays, *H pylori* was identified as a major infectious agent as a vital player in the pathogenesis of ACS and could increase risk of ACS through multiple mechanisms. It is well known that chronic infections are accompanied by a persistent inflammatory response. Several studies reported that *H pylori* infection could increase serum levels of fibrinogen and inflammatory cytokines, which led to a systemic inflammatory response.^[53,54]^ On one hand, The inflammatory cytokines may contribute to the instability of the plaque by increasing the production of oxide free radical and proteolytic enzymes. On the other hand, the inflammatory cytokines could also promote an inflammatory response in arteries and damage vascular walls.^[55,56]^ The systematic inflammatory response, accompanying with an increase in plasma fibrinogen and inflammatory cytokines, could accelerate the atherosclerotic plaque instability and plaque disruption.^[54]^ In addition, *H pylori* also could induce lipid peroxidation and oxidized LDL, which played an important role in both of early development and late evolution of atherosclerotic lesions.^[57,58]^

In the meta-analysis, we could more accurately clarify the relationship between *H pylori* and ACS with a larger number of samples. We not only improved the inclusion and exclusion criteria, but also evaluated the quality of all included studies. Sensitivity analysis and publication bias were conducted in the meta-analysis, which all suggested that pooled results were stable and reliable. Therefore, our results could most accurately describe the association between ACS and *H pylori*. However, several limitations should be considered in this meta-analysis. First, it is possible that we may omit several relevant publications though systematic search was performed. Second, Africa is the world's highest *H pylori* prevalence region (79.1%) in the world.^[50]^ However, no African study was included in the meta-analysis, which may obscure any potential association. Finally, heterogeneity could not be eliminated in our meta-analysis. The diagnostic method in detecting *H pylori* infection could affect the accuracy and creditability of the data, which may be the source of heterogeneity. Ninety-five percent included studies detected *H pylori* infection by ELISA; however, different ELISA kits provided different sensitivity and specificity. In the meta-analysis, only 3 included studies used the same ELISA kit to detect *H pylori* infection.^[14,25,48]^ The pooled result also showed a significant association between *H pylori* and ACS (OR = 2.27, 95% CI 1.72–4.31, *P*_*H*_ = .410) in the same ELISA kit subgroup and the heterogeneity decreased markedly from 84.9% to 0%, which suggested that the detection method of *H pylori* contributed to the source of heterogeneity. In addition, the accuracy of serological testing for *H pylori* were descend by the increase of age.^[59]^ Thus, *H pylori* infection should be detected by a unified method in the future, such as ^13^C-urea breath test, which may decrease the heterogeneity.

In conclusion, our meta-analysis suggested that *H pylori* infection was associated with an increased risk of ACS, especially in developing countries. *H pylori* is easily screened and can be treated with a wide range of drugs. Thus, more high-quality and well-designed studies are needed to confirm whether the treatment of *H pylori* is an effective way to reduce ACS risk.

## Author contributions

**Conceptualization:** Yizhen Fang, Huabin Xie.

**Data curation:** Yizhen Fang, Chunming Fan, Huabin Xie.

**Formal analysis:** Yizhen Fang, Chunming Fan, Huabin Xie.

**Investigation:** Yizhen Fang, Chunming Fan, Huabin Xie.

**Methodology:** Yizhen Fang, Chunming Fan, Huabin Xie.

**Project administration:** Yizhen Fang, Chunming Fan.

**Resources:** Yizhen Fang, Chunming Fan, Huabin Xie.

**Software:** Yizhen Fang, Chunming Fan, Huabin Xie.

**Supervision:** Huabin Xie.

**Validation:** Huabin Xie.

**Visualization:** Chunming Fan, Huabin Xie.

**Writing – original draft:** Yizhen Fang.

**Writing – review and editing:** Yizhen Fang.
